# Neurodifferentiation and Neuroprotection Potential of Mesenchymal Stromal Cell-Derived Secretome Produced in Different Dynamic Systems

**DOI:** 10.3390/biomedicines11051240

**Published:** 2023-04-22

**Authors:** Cláudia Raquel Marques, Miguel de Almeida Fuzeta, Raquel Medina dos Santos Cunha, Joana Pereira-Sousa, Deolinda Silva, Jonas Campos, Andreia Teixeira-Castro, Rui Amandi Sousa, Ana Fernandes-Platzgummer, Cláudia L. da Silva, António José Salgado

**Affiliations:** 1 Life and Health Sciences Research Institute (ICVS), School of Medicine, University of Minho, 4710-057 Braga, Portugal; 2 ICVS-3Bs PT Government Associate Laboratory, 4710-057 Braga/Guimarães, Portugal; 3 Department of Bioengineering and iBB-Institute for Bioengineering and Biosciences, Instituto Superior Técnico, Universidade de Lisboa, 1049-001 Lisboa, Portugal; 4 Associate Laboratory i4HB-Institute for Health and Bioeconomy, Instituto Superior Técnico, Universidade de Lisboa, 1049-001 Lisboa, Portugal; 5 Stemmatters, Biotecnologia e Medicina Regenerativa S.A., 4805-017 Barco, Portugal

**Keywords:** bioreactor, dynamic systems, mesenchymal stromal cells, Parkinson’s disease, secretome

## Abstract

Parkinson’s disease (PD) is the second most common neurodegenerative disorder and is characterized by the degeneration of the dopamine (DA) neurons in the substantia nigra pars compacta, leading to a loss of DA in the basal ganglia. The presence of aggregates of alpha-synuclein (α-synuclein) is seen as the main contributor to the pathogenesis and progression of PD. Evidence suggests that the secretome of mesenchymal stromal cells (MSC) could be a potential cell-free therapy for PD. However, to accelerate the integration of this therapy in the clinical setting, there is still the need to develop a protocol for the large-scale production of secretome under good manufacturing practices (GMP) guidelines. Bioreactors have the capacity to produce large quantities of secretomes in a scalable manner, surpassing the limitations of planar static culture systems. However, few studies focused on the influence of the culture system used to expand MSC, on the secretome composition. In this work, we studied the capacity of the secretome produced by bone marrow-derived mesenchymal stromal cells (BMSC) expanded in a spinner flask (SP) and in a Vertical-Wheel™ bioreactor (VWBR) system, to induce neurodifferentiation of human neural progenitor cells (hNPCs) and to prevent dopaminergic neuron degeneration caused by the overexpression of α-synuclein in one *Caenorhabditis elegans* model of PD. Results showed that secretomes from both systems were able to induce neurodifferentiation, though the secretome produced in the SP system had a greater effect. Additionally, in the conditions of our study, only the secretome produced in SP had a neuroprotective potential. Lastly, the secretomes had different profiles regarding the presence and/or specific intensity of different molecules, namely, interleukin (IL)-6, IL-4, matrix metalloproteinase-2 (MMP2), and 3 (MMP3), tumor necrosis factor-beta (TNF-β), osteopontin, nerve growth factor beta (NGFβ), granulocyte colony-stimulating factor (GCSF), heparin-binding (HB) epithelial growth factor (EGF)-like growth factor (HB-EGF), and IL-13. Overall, our results suggest that the culture conditions might have influenced the secretory profiles of cultured cells and, consequently, the observed effects. Additional studies should further explore the effects that different culture systems have on the secretome potential of PD.

## 1. Introduction

Mesenchymal stromal cells (MSC) are a population of multipotent progenitor cells with the ability to self-renew and the potential to differentiate into different cell lines, and that can be retrieved from different tissues [1,2]. Despite the advantages and limitations of each tissue source, bone marrow is still the gold standard and most widely used tissue source. Gathered evidence suggests that the beneficial effects of the secretome of MSC are equivalent to the effects of the administration of MSC and extend to different domains, including the hepatic, skeletal, cardiovascular, and nervous systems, among others (reviewed in [3]). The benefits of secretome have been studied for a large spectrum of conditions of the nervous system, including Parkinson’s disease (PD), where it showed to outperform MSC transplantation-related approaches [4,5,6,7].

PD is characterized by the decrement in dopamine (DA) levels due to the degeneration of dopaminergic cells present in the substantia nigra pars compacta (SNpc) [8]. The molecular pathways responsible for neurodegeneration in PD are not well clarified, but accumulated evidence suggests that mitochondrial dysfunction is highly linked to the development of the disease. This pathway leads to oxidative stress, accumulation of oxidized dopamine, which is correlated with lysosomal dysfunction, as well as alpha-synuclein (α-synuclein) accumulation and aggregation [8,9]. Specifically, α-synuclein is a protein mainly located at the pre-synaptic terminal that is supposed to be involved in vesicular packaging, trafficking, and synaptic transmission [10]. However, the accumulation of α-synuclein and the presence of oligomerized or aggregated forms of α-synuclein can greatly impact different cellular mechanisms, leading to oxidative stress and neurodegeneration [11].

Different disease-modifying strategies for PD have been studied, including the use of secretome from MSC, due to its neuroprotective and neurodifferentiation potential. In fact, MSC-derived secretome has protected cultured rodent cortical neurons from death, a process that was dependent on the phosphoinositide 3-kinase/protein kinase B survival pathway. Brain-derived neurotrophic factor (BDNF) was defined as particularly responsible for the neuroprotective effect [12]. The same factor is required for the existence of an adequate number of DA neurons in the SNpc [13]. Moreover, our group has been showing the beneficial effects of secretomes from MSC on the survival of DA neurons and stimulation of neurodifferentiation in different models of the disease [6,14,15,16].

Bioreactors are an important platform to enable the establishment of dynamic cultures, that better recreate the microenvironment of MSC [17]. As already shown by our group, the production of secretomes in a bioreactor system can impact the expression of different factors and even enhance the expression of others, compared with secretomes produced in static conditions [15,18]. Bioreactors also provide the means to produce large volumes of secretome, fundamental for application in large groups of patients. Nevertheless, there is a wide range of bioreactor configurations available in the market, with different characteristics, and the effects of their particularities in the secretory profile of MSC are poorly understood.

In this work, two different mechanically agitated systems were used to produce secretomes from bone marrow-derived MSC (BMSC). The widely known spinner flask (SP) system has a cylindrical shape and is harnessed with 90° paddles and a magnetic stir bar. Its agitation mechanism greatly contrasts with the Vertical-Wheel™ bioreactor (VWBR) in which agitation is generated by a large vertical impeller and a U-shaped bottom, providing mixing and suspension of particles with low agitation speeds [19]. Both systems are scalable and are available in a single-use format, which could facilitate translation into clinical use. Indeed, single-use systems are important for biopharmaceutical manufacturing, to eliminate the need for cleaning and sterilization between runs, thus significantly reducing the contamination rates and production costs [20]. Herein, the capacity of MSC-derived secretomes produced in both dynamic systems to induce neurodifferentiation and neuroprotection was assessed and compared.

## 2. Materials and Methods

### 2.1. Human Bone Marrow Mesenchymal Stromal Cell (BMSC) Cultures

BMSCs used in this study are part of the cell bank available at the Stem Cell Engineering Research Group (SCERG), iBB-Institute for Bioengineering and Biosciences at Instituto Superior Técnico (IST). MSCs were previously isolated/expanded according to protocols previously established at iBB-IST) [21]. Originally, BMSCs were isolated from bone marrow aspirates obtained from healthy donors after written informed consent at Instituto Português de Oncologia Francisco Gentil, Lisboa, Portugal, according to the Directive 2004/23/EC of the European Parliament and of the Council of 31 March 2004 on setting standards of quality and safety for the donation, procurement, testing, processing, preservation, storage and distribution of human tissues and cells (Portuguese Law 22/2007, 29 June), with the approval of the Ethics Committee of the respective clinical institution, according to the Portuguese Regulation (Law 21/2014, 16 April). MSCs were retrieved according to the established protocols as described by dos Santos and colleagues [21]. Cells from one donor with 4 and 5 passages were used.

### 2.2. BMSC Cultures under Static Conditions

Cryopreserved BMSCs were thawed and plated at a cell density of 3000 cells·cm^−2^, on T-75 flasks with low glucose (1 g·L^−1^) Dulbecco’s Modified Eagle Medium [DMEM, (Gibco, Thermo Fisher Scientific, New York, NY, USA) supplemented with 5% *v*/*v* human platelet lysate (hPL) UltraGRO™-PURE (AventaCell, Atlanta, GA, USA) and Antibiotic-Antimycotic (1×) (Gibco, Thermo Fisher Scientific) (DMEM/HPL). Alternatively, cells were plated at the same cell density in CELLstart™ substrate (Gibco, Thermo Fisher Scientific) pre-coated T-75 flasks with StemPro™ MSC SFM XenoFree medium (Gibco, Thermo Fisher Scientific) supplemented with Glutamax (1×) (Gibco, Thermo Fisher Scientific) and Antibiotic-Antimycotic (1%) (StemPro). Cells cultured in DMEM-HPL were grown in VWBR, whereas cells grown in SP were cultured in StemPro. At 70% cell confluence, MSCs were harvested with 1× TrypLE™ Select Enzyme solution (Gibco, Thermo Fisher Scientific) for 5 min at 37 °C. Cell number and viability were determined using the Trypan Blue (Gibco, Thermo Fisher Scientific) exclusion method.

### 2.3. Microcarrier Preparation

Animal product-free SoloHill plastic microcarriers [MCs (Sartorius, Gottingen, Germany)] of 360 cm^2^·g^−1^ superficial area, were used for BMSC culture in both systems. The preparation of the MCs varied according to the culture medium used in each system.

#### 2.3.1. Inoculation of the Vertical-Wheel Bioreactor (VWBR)

Following sterilization by autoclaving (120 °C, 20 min), the coating of MCs with a solution of DMEM supplemented with 50% hPL UltraGRO™-PURE was performed using a Thermomixer^®^ comfort (Eppendorf AG, Hamburg, Germany) following a protocol consisting of cycles of 2 min at 750 rpm agitation and 10 min without agitation, during 1 h. Prior to cell inoculation, MCs were resuspended in DMEM/HPL [22].

#### 2.3.2. Inoculation of the Spinner Flask (SP)

After sterilization by autoclaving (120 °C, 20 min), MCs were coated with a CELLstart™ substrate (diluted 1:100 in 1× PBS) for 1 h at 37 °C, with an intermittent agitation (cycles of 2 min at 750 rpm, 8 min without agitation) using a Thermomixer^®^ comfort, and afterwards equilibrated in pre-warmed StemPro [23].

### 2.4. BMSC Culture in VWBR

Expansion of BMSC in VWBR was generally performed as previously described [22]. In summary, PBS MINI vertical-wheel bioreactor, namely PBS 0.1 (PBS Biotech, Camarillo, CA, USA) was operated at its full working volume (100 mL). Previously prepared MCs were used at a concentration of 20 g·L^−1^. Following the introduction of the MCs into the bioreactor, BMSC previously expanded under static conditions for 2 passages, were transferred to the bioreactor (5 × 10^6^ cells). DMEM/HPL was added to reach 60 mL of culture medium inside the bioreactor. The culture was maintained at 37 °C and 5% CO_2_. During the first 6 h, the agitation was set to cycles of 25 rpm for 1 min, followed by 20 min without agitation. At the end of this regime, the agitation was set to 25 rpm. After 48 h, 40 mL of fresh culture medium with a glucose pulse (3 g/L) was added to the VWBR, to achieve a final volume of 100 mL. Feeding was performed from the third day of culture on a daily basis, by replacing 25% of volume with fresh culture medium supplemented with a glucose pulse (3 g/L).

Daily sampling of the culture was performed to determine total cell number and metabolite concentration. Briefly, when the MCs settled, supernatant was harvested, centrifuged, transferred to a new tube, and stored at −20 °C until further analysis of glucose and lactate concentrations. Afterwards, a representative sample of the homogenized culture was collected, and the MCs were incubated with TrypLE™ Select Enzyme solution at 37 °C for 7 min and 750 rpm, using a Thermomixer^®^ comfort. After stopping the reaction by diluting with culture medium, mechanical dissociation was performed by pipetting up and down and the mixture was then filtered using a 100 µm cell strainer (BD Biosciences, Franklin Lakes, NJ, USA) to remove the MCs. Cells were centrifuged at 350× *g* for 7 min, and total cell number and viability were determined throughout time using the Trypan Blue exclusion method. The determination of the specific growth rate was based on an exponential fitting to the experimental data that correspond to the exponential growth phase. The doubling time was calculated by the ratio between the natural logarithm of 2 and the growth rate. Adhesion efficiency to the MCs was calculated as the ratio between the total number of cells on day 1 and the number of inoculated cells.

To monitor cell adhesion and growth within the culture, an additional sample was collected daily. Following cell fixation using 4% paraformaldehyde (PFA; Sigma-Aldrich, St. Louis, MO, USA), MCs containing cells were incubated with 4-6-diamidino-2-phenylindole-dihydrochloride (DAPI, Sigma-Aldrich) at 1.5 mg·L^−1^ for 10 min and observed in a fluorescence inverted microscope (Leica DMI 3000 B).

### 2.5. BMSC Culture in SP

Bellco^®^ spinner flasks (Bellco Glass, Vineland, NJ, USA) offer a working volume of 100 mL but were operated at 80 mL. Prior to use, spinner flasks were autoclaved and treated with Sigmacote^®^ (Sigma-Aldrich) to prevent microcarrier adhesion to the surface of the flask. Notably, 20 g·L^−1^ MCs, prepared following the aforementioned protocol, and 4 × 10^6^ MSC previously expanded under static conditions with StemPro for 2 passages, were added to the SP with a total volume of 40 mL and incubated at 37 °C and 5% CO_2_. Following cell inoculation, agitation was set to 30 rpm. After 24 h, agitation was increased to 40 rpm. The necessary volume of medium to attain 80 mL was added 96 h post-inoculation. Feeding was performed from the fourth or fifth day of culture onwards, by replacing 25% of the volume with pre-warmed medium or by the addition of a glucose pulse to maintain the levels of glucose at a non-limiting concentration (superior to 1 mM) [24].

Daily sampling was performed according to the methodology followed for the culture in the VWBR. Adhesion, growth rate, and population doubling time were calculated for both cultures.

### 2.6. BMSC Conditioning and Secretome Collection

BMSCs were cultured in both dynamic cultures for 24 h before the end of the dynamic cultures. The culture medium was totally replaced by fresh medium for conditioning. For that, the exhausted medium was removed, and cell-containing MC systems were washed once with 100 mL of Neurobasal-A (Gibco, Thermo Fisher Scientific) or AlphaMEM (Gibco, Thermo Fisher Scientific) culture medium. Afterwards, 100 mL for the VWBR or 80 mL for the SP of AlphaMEM (SP1 and VWBR1) or Neurobasal-A (SP2 and VWBR2) medium, with 1% Antibiotic-Antimycotic were added to the culture systems. After 24 h, the medium was harvested and centrifuged at 300× *g* for 10 min to remove cell debris.

The secretome collected using AlphaMEM medium was intended to be used for *C. elegans* assays and was concentrated by centrifugation to 2× using a Vivaspin 5 kDa cut-off concentrator (GE Healthcare, UK). The secretome collected in Neurobasal-A medium was used for the differentiation of human neural progenitor cells (hNPCs) and was not concentrated. Aliquots of secretome were flash-frozen with liquid nitrogen and stored at −80 °C.

### 2.7. Metabolite Analysis throughout Cultures

The consumption of glucose and the production of lactate were monitored throughout the culture period. Upon medium exchange, the concentration of both metabolites was assessed before and after medium replacement. Glucose and lactate concentrations were analyzed using a YSI 7100MBS equipment (YSI Incorporated, Yellow Springs, OH, USA). The yield of lactate from glucose (YLac/Glc), was calculated for each day as the ratio between the specific metabolic rates, qmet (mol·day^−1^·cell^−1^), which corresponds to the production of lactate during that day and the consumption of glucose during the same period. These parameters were determined as described in the literature [25].

### 2.8. Immunophenotypic Analysis of BMSC

At the end of the culture, cells were harvested from the MCs and analyzed for the expression of specific surface antigens by flow cytometry. Approximately 1 × 10^5^ cells were resuspended in 1× PBS and incubated for 15 min in the dark with the antibodies. After a washing step with 1× PBS, cells were fixed with 1% PFA and stored at 4 °C until analysis using a BD FACSCaliburTM platform, equipped with the CellQuestTM software (BD Biosciences). The antibodies used were CD105 PE (phycoerythrin), CD90 FITC (Fluorescein isothiocyanate), CD80 PE, CD73 PE, CD45 FITC, CD34 FITC, CD14 PE, and HLA-DR PE. All of them were purchased from BioLegend (USA), except for CD105 PE (Thermo Fisher Scientific, USA). Non-stained cells were also prepared for every experiment. A minimum of 10,000 events were collected for each sample. Analysis was performed using FlowJo™ v10 software (BD Biosciences). The employed gating strategy can be found in the supplementary data.

### 2.9. Multilineage Differentiation Assays of BMSC

Upon dynamic culture, multilineage differentiation assays were performed for BMSC as previously described [26]. Briefly, BMSCs were retrieved from the MCs (as previously described) and cultured with appropriate medium [adipogenesis, osteogenesis, or chondrogenesis StemPro differentiation kits (Thermo Fisher Scientific)] for 22 days, in order to stimulate the differentiation into each one of three lineages. Differentiation toward an adipocytic phenotype was assessed through staining based on the Oil Red-O solution to visualize the existence of lipidic vacuoles. For osteogenic differentiation, cells were prepared for alkaline phosphatase (ALP) and von Kossa staining. ALP enables the visualization of osteogenic progenitors and von Kossa stains calcium deposits. For chondrogenic differentiation, Alcian Blue was used to stain the proteoglycan aggrecan (an indicator for cartilage formation), which is dark blue.

### 2.10. Expansion of Human Neural Progenitor Cells and Incubation with hBMSC Secretome

The procedure for isolation of hNPCs was already described by our group and followed the strict ethical guidelines established and approved by the Conjoint Health Research Ethics Board (CHREB, University of Calgary, AB, ID: E-18786) [27]. hNPCs were thawed and placed onto a T-75 flask containing 10 mL of Complete NeuroCultTM-NS-A Proliferation Medium (STEMCELL Technologies, Canada). After two days, cells were harvested and mechanically dissociated into a single-cell suspension and plated at a density of 1 × 10^4^ viable cells/cm^−2^ in new culture flasks. The feeding regime was performed every 2 days, by adding 10% of fresh complete medium. After 10–12 days of culture, coverslips (Marienfeld, Germany) were placed inside a 24-well plate and were pre-coated with poly-D-lysine hydrobromide (100 μg/mL) (Sigma-Aldrich, USA) for 2 h at room temperature (RT) and laminin (10 μg/mL) (Sigma-Aldrich) for 3 h at 37 °C. hNPCs were mechanically dissociated and plated at a density of 5 × 10^4^ cells/cm^−2^. Cells were incubated at 37 °C, 5% CO_2_, 95% air, and 90% relative humidity for 5 days with secretome collected in Neurobasal-A medium from each culture system (SP2 and VWBR2) supplemented with 1% GlutaMAX (Gibco). Neurobasal-A medium with 1% Antibiotic-Antimycotic and 1% GlutaMAX was used as a control.

### 2.11. Immunocytochemistry Analysis of Human Neural Progenitor Cells

Following incubation with secretome, the induction of differentiation was evaluated. Therefore, cells were fixed with 4% PFA (PanReac, Barcelona), washed with 1× PBS, and blocked as already described [28]. Following blocking, cells were incubated for 1 h at RT with the primary antibodies, namely, rabbit anti-doublecortin (DCX; 1:300, Abcam, USA), to detect immature neurons and mouse microtubule-associated protein-2 (MAP-2; 1:500, Sigma) and to detect mature neurons. After a washing step, cells were incubated for 1 h at RT with secondary antibodies (1:1000): Alexa Fluor 488 goat anti-rabbit (Thermo Fisher Scientific) and Alexa Fluor 594 goat anti-mouse (Thermo Fisher Scientific). Following this procedure, DAPI (Life Technologies) was added for 5 min. Coverslips were observed under an Olympus BX-61 Fluorescence Microscope (Olympus, Tokyo, Japan). Briefly, four coverslips per condition and ten representative fields per coverslip were analyzed, and the experiment was repeated independently four times. Results are presented as percentage of cells positive for MAP-2 or DCX markers divided by the total number of cells/field (DAPI-positive cells).

### 2.12. Nematode Strains and Culture Conditions

All strains were kept in nematode growth media (NGM) containing agar plates seeded with *Escherichia coli* OP50 at 20 °C, as previously described [29]. Strain BZ555 *egIs1* (P*dat-1*::green fluorescent protein [GFP]) was acquired from the Caenorhabditis Genetics Center. Strain UA44 (*baInl1*; P*dat-1*::α-synuclein high, P*dat-1*::GFP) was gently provided by Guy Caldwell (University of Alabama).

Secretome collected in AlphaMEM medium was used for *C. elegans* assays. Briefly, three types of plates were prepared: two were seeded with 2x concentrated secretome in AlphaMEM from VWBR or SP diluted in inactivated OP50 (secretome final concentration = 1×) and the other was seeded with 2× concentrated AlphaMEM diluted in inactivated OP50 (AlphaMEM final concentration = 1×) as control. To prepare inactivated OP50, bacteria were grown overnight at 37 °C and 150 rpm in Luria Broth medium, pelleted by centrifugation, inactivated by three cycles of freeze/thawing, frozen at −80 °C and then resuspended in S-medium supplemented with 25 U/mL PenStrep (Thermo Fisher Scientific) and 50 U/mL Nystatin (Sigma-Aldrich).

### 2.13. Quantitative Analysis of Dopaminergic Neuronal Loss

Age-synchronized BZ555 and UA44 worms were obtained by egg-laying, by plating adult worms in freshly prepared plates followed by their removal from plates after 2 h (day 0). Worms born on the treated plates were maintained until day 10 and prepared for scoring of dopaminergic neurons according to the procedures described by Marques and colleagues [30]. Intact dopaminergic neurons were scored, and the experiment was repeated independently three times (*n* = 12 animals/condition).

### 2.14. Membrane Antibody Arrays

Some soluble factors present in all produced secretomes were identified and quantitatively compared using Human Cytokine Antibody Array 5 (AAH-CYT-5, RayBiotech, Peachtree Corners, GA, USA) and Human Neuro Discovery Array C1 (AAH-NEU-1, RayBiotech). Each antibody array matrix can simultaneously detect 80 cytokines and 20 neurologically relevant molecules, respectively. Briefly, antibody arrays were incubated with all four secretomes overnight at 4 °C. Membranes were then processed according to the manufacturer’s instructions. Relative expression levels were evaluated by comparing signal intensities, which were obtained with the Sapphire Biomolecular Imager (Azure Biosystems, Dublin, OH, USA) and quantified by densitometry. Membranes present a positive control that was used to normalize the results from the different membranes being compared, which resulted in a normalized intensity value corresponding to each factor.

### 2.15. Statistics

Statistical analysis was performed using the IBM SPSS statistics 25 software. The normal distribution of continuous variables was analyzed according to Shapiro-Wilk or Kolmogorov-Smirnov normality tests. Homogeneity of variances was assessed using Levene’s test. When both assumptions were not met, a robust ANOVA with Welch correction and bootstrap with BCA were performed. Bootstrap sampling was followed by one-way ANOVA with Sidak post hoc test with bias correction (for neurodifferentiation and neuroprotection assays). Pearson’s chi-squared test was used for proportion analysis, with the follow-up z-test for independent proportions with the Bonferroni correction (neuroprotection assay). Appropriate effect size measures were used for each test (ω2p for ANOVA with Welch correction and V for Pearson’s chi-square test). Detailed statistics are available in Supplementary Table S1.

## 3. Results

### 3.1. BMSCs Were Successfully Expanded in Both Dynamic Systems

**Figure 1:**
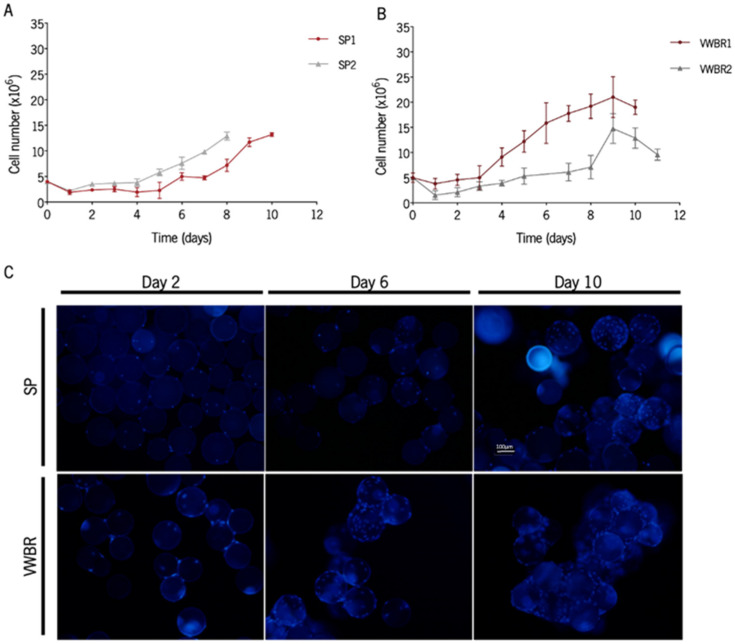
Ex vivo expansion of BMSC in the two dynamic culture systems. Representation of cell growth for cultures in the (**A**) SP and in the (**B**) VWBR. SP1 and VWBR1 represent the secretomes collected in AlphaMEM medium and SP2 and VWBR2 represent the secretomes collected in Neurobasal-A medium. Results are presented as mean ± SD of cell count for each time point. (**C**) Representative images of BMSC attached to MCs at 2, 6, and 10 days of culture in SP and VWBR. Cell nuclei were stained with DAPI and images were acquired using a fluorescence microscope. Scale bar = 100 μm. SP, Spinner flask system; VWBR, Vertical-Wheel™ bioreactor; SD, standard deviation.

In this study, we employed each one of the culture systems, making use of previously established and optimized culture conditions aimed at maximizing cell expansion [22,23,31,32,33]. The cells were successfully expanded in both systems (Figure 1A,B). The percentage of cells that adhered to the MCs within the first 24 h was also determined. Both the maximum and the minimum values for cell adhesion efficiency were obtained with VWBR, but the average of both systems is similar (Table 1). The highest fold expansion (4.19 ± 0.81) was obtained with the VWBR (VWBR1). MC colonization increased over time with MSC expansion, and the higher occupancy of MCs translated into a boost in MC aggregation (Figure 1C). The highest specific growth rates and the lowest doubling times were attained in both cultures with SP; the VWBR2 culture had the lowest growth rate, probably as a result of the low initial adhesion efficiency (31% versus 76% for VWBR1, 48% for SP1, and 55% for SP2).

### 3.2. Glycolitic Metabolism Is Kept Consistent among Culturing Systems

**Figure 2:**
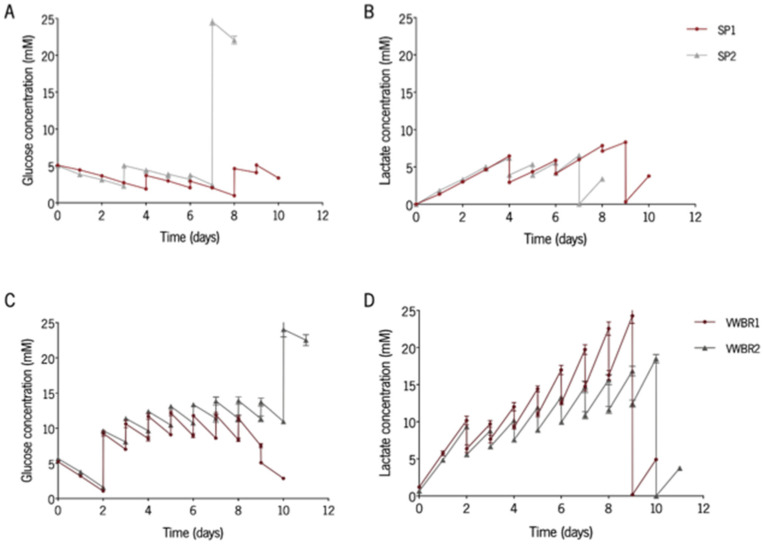
Metabolic analyses of the expansion of BMSC in the SP and VWBR. Concentration profiles of glucose and lactate during the culture in (**A**,**B**) SP and in (**C**,**D**) VWBR. SP1 and VWBR1 represent the secretomes collected in AlphaMEM medium and SP2 and VWBR2 represent the secretomes collected in Neurobasal-A medium. Results are presented as mean ± SD of two independent readings.

The determination of nutrient consumption and production of metabolites is a relevant procedure to ascertain the availability of nutrients or accumulation of waste products. Therefore, the concentration of glucose and lactate was monitored daily (Figure 2A–D). Glucose and lactate profiles were similar between both cultures in each system. The lowest values for glucose concentration were detected in SP cultures (Figure 2A,C). The levels of glucose in all cultures were kept within non-limiting (over 1 mM) levels for cell proliferation (Figure 2A,C). [24]. For all cultures, the highest values for lactate concentration were registered during the last days before the conditioning day and were always below 35 mM, described in the literature as a threshold inhibitory lactate concentration (Figure 2B,D) [24]. The evaluation of the consumption of glucose and production of lactate enables the determination of the average yield of lactate from glucose (Y_Lac/Glc_). For all cultures, the value of Y_Lac/Glc_ was approximately 2 mol lactate·mol^−1^ glucose (Supplementary Figure S1), which is a typical value when cells rely on glycolysis for energy metabolism [34].

### 3.3. Cells Expanded in Both Systems Retain MSC Phenotype and Are Capable of Multilineage Differentiation

**Figure 3:**
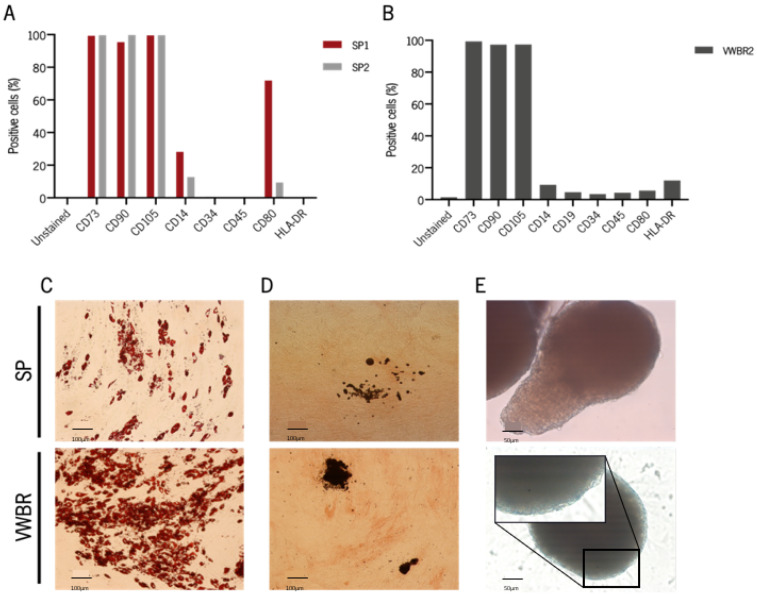
Immunophenotypic analysis and multilineage differentiation potential of BMSC after expansion under dynamic conditions. The percentage of expression of each surface antigen (CD14, CD19, CD34, CD45, CD73, CD80, CD90, CD105, and HLA-DR), analyzed by flow cytometry is represented for one of the cultures in the (**A**) SP and (**B**) VWBR. Representative images of multipotency characterization of BMSC cultured in SP and VWBR through multilineage differentiation assays, upon 22 days under (**C**) Oil red-O for adipogenic, (**D**) alkaline phosphatase (ALP) and von Kossa staining for osteogenic, and (**E**) Alcian Blue for chondrogenic differentiating conditions. Scale bar = 100 μm. SP, Spinner flask system; VWBR, Vertical-Wheel™ bioreactor.

Following BMSC expansion, immunophenotypic assays were performed in order to guarantee that their immunophenotype was not affected by the culture in each of the dynamic systems (Figure 3A and Figure S2) [35]. Following culture in SP, CD73, CD90, and CD105 biomarkers were expressed in more than 95% of the cells (Figure 3A and Figure S2). Regarding MSC “negative” markers, we observed that a considerable percentage of cells expressed CD14 and CD80, especially cells from the first culture in SP. Overall, cells from VWBR1 kept the characteristic MSC immunophenotype after culture in the VWBR system (Figure 3B and Figure S2). Moreover, cells cultured in both systems preserved the multilineage differentiation ability toward adipogenic (Figure 3C), osteogenic (Figure 3D), and chondrogenic (Figure 3E) lineages.

### 3.4. Secretomes Produced in Both Dynamic Culture Systems Were Able to Induce Neurodifferentiation

**Figure 4:**
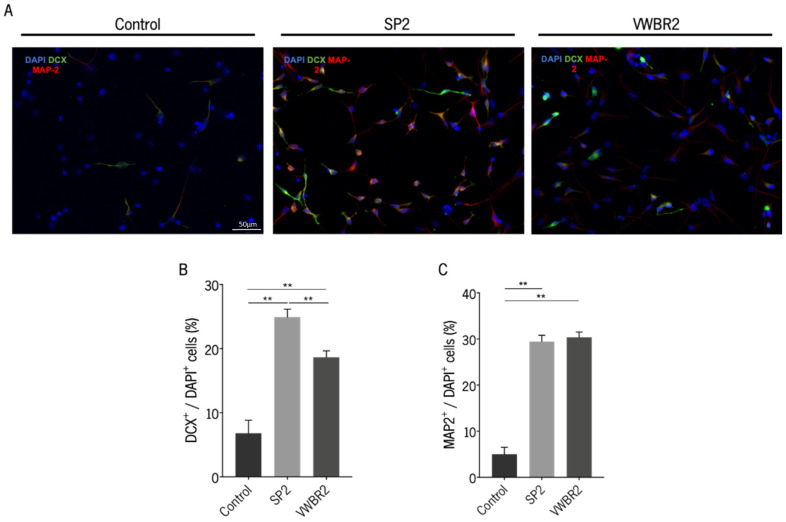
Secretomes collected in SP and VWBR were able to increase the survival and differentiation of hNPCs. In vitro differentiation of hNPCs was assessed by counting immature (DCX^+^ cells) and mature (MAP-2^+^ cells) neurons and dividing by the total number of cells (DAPI^+^). (A) Representative photographs for each condition showing cells positive for DCX and/or MAP-2. Scale bar = 50 μm. Graphical representation of the percentage of cells expressing (**A**) DCX and (**B**) MAP-2. SP2 and VWBR2 represent the secretomes collected in Neurobasal-A medium. A total of four coverslips per condition and ten representative fields per coverslip were analyzed, and the experiment was repeated independently four times. ** *p* < 0.01 (ANOVA, SIDAK test corrected for BCA). Control group (Neurobasal-A + 1% l-glutamine). DAPI, 4-6-diamidino-2-phenylindole-dihydrochloride; DCX, doublecortin; MAP-2, microtubule-associated protein-2.

The capacity of SP2 and VWBR2 secretomes to induce neurodifferentiation was explored by incubating hNPCs with both secretomes, followed by the analysis of the expression of DCX and MAP-2. After the incubation period, both secretomes induced cells toward a differentiated state, which was confirmed by the expression of both markers (Figure 4A,B). We observed that the SP2 secretome had a superior effect regarding the expression of DCX, an early neuronal marker, compared with the VWBR2 secretome and the positive control [36]. The same was not seen for MAP-2 expression because both secretomes induced the same level of MAP-2 expression (a characteristic marker of mature neurons) [37].

### 3.5. Distinct Capacity to Induce Neuroprotection Was Shown with Secretomes from Different Systems

**Figure 5:**
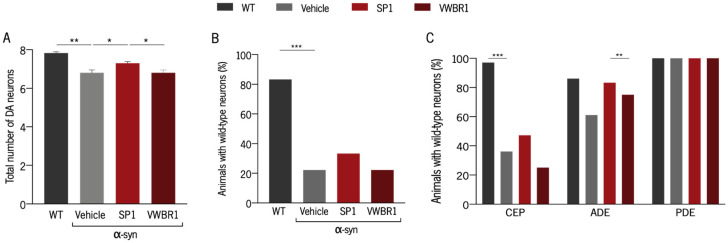
BMSC secretome produced using the SP protects dopaminergic neurons from α-synuclein-associated effects. (**A**) Graphical representation of the total number of intact dopaminergic neurons for each condition (ANOVA, SIDAK test corrected for BCA). (**B**) The proportion of animals with intact DA neurons for each condition was determined by counting the number of animals with WT neurons. (**C**) The proportion of animals with intact CEP, ADE, or PDE neurons was determined by counting the animals with WT DA neurons belonging to each subpopulation (Pearson’s chi-square test). SP1 and VWBR1 represent the secretomes collected in AlphaMEM medium. A total of 36 animals were assayed per group across three independent experiments. * *p* < 0.05, ** *p* < 0.01 *** *p* < 0.001. Vehicle is the Alpha-MEM medium. ADE, anterior deirid; CEP, cephalic; DA, dopamine; PDE, posterior deirid; WT, wild-type.

The secretomes SP1 and VWBR1 were applied to a *C. elegans* model that overexpresses WT α-synuclein in their eight dopaminergic neurons. This model of PD is characterized by an age-dependent loss of dopaminergic neurons that can be quantified microscopically due to the expression of GFP in the same cells. Eggs from adult *C. elegans* were plated in Petri dishes containing secretome diluted in their food source, and after 10 days, DA neurons were scored. We observed that only animals treated with secretome produced in the SP were less susceptible to the effects of α-synuclein because they presented a higher number of intact DA neurons (Figure 5A). When we compared the percentage of animals with WT DA neurons, the same tendency was observed, though there were no statistically significant differences between groups (Figure 5B). Lastly, the differences between groups regarding the three populations of DA neurons [cephalic (CEP), anterior deirid (ADE), and posterior deirid (PDE)] were analyzed. Animals treated with SP1 showed a higher number of ADE neurons when compared with animals treated with VWBR1 secretome (Figure 5C). Both groups clearly displayed a higher number of ADE neurons compared with untreated animals, but this difference was not statistically significant.

### 3.6. Cells Cultured in Both Systems Had Distinct Secretory Profiles

**Figure 6:**
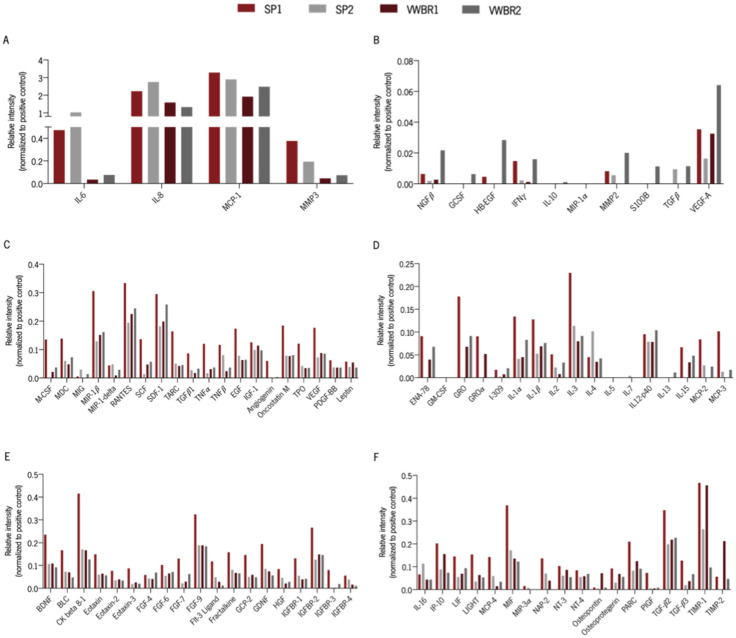
(**A**–**F**). Human antibody array identification of neurologically relevant proteins and cytokines in the secretomes from both dynamic systems. Antibody arrays were incubated with secretomes produced in SP and VWBR and collected in AlphaMEM (SP1 and VWBR1) and Neurobasal-A (SP2 and VWBR2) medium. Signal intensity of arrays was analyzed by densitometry, and the relative intensities of individual proteins were calculated after normalizing to the positive controls on each array. The experiment was performed once for each secretome.

The expression of specific factors was compared among the different secretomes, to depict possible players that can be contributing to the observed effects. Antibody arrays were incubated with secretome, and the analysis of the signal intensity enabled the determination of the relative intensity of each factor (Figure 6A–F). Most of the molecules assessed presented higher relative intensities in the SP1 secretome. Two molecules were clearly highly expressed in all secretomes, namely the chemokines Interleukin 8 (IL-8) and Monocyte Chemoattractant Protein-1 (MCP-1) (Figure 6A). The relative intensities of IL-6, MMP3, and TNF-β were higher in both secretomes produced in SP, whereas osteopontin relative intensity revealed a higher value in VWBR1 secretome and NGFβ, GCSF, HB-EGF, and IL-13 in VWBR2.

## 4. Discussion

The development of a protocol for the large-scale production of clinical-grade secretome is an important step towards the implementation of secretome-based therapies. Bioreactors are a valuable tool for the scalable production of cell-based products, namely, secretomes. Moreover, these can be implemented not only for secretome production but also as a preconditioning strategy for the functional enhancement of MSC features and their secreted factors [15,18,38].

In this work, two different dynamic systems were used to culture BMSC and collect their secretome. A microcarrier-based culture system was implemented in an SP and in a VWBR. The SP consists of a cylindrical flask with a small horizontally rotating paddle impeller which requires an increased rotation to improve homogenization, which implies higher shear stress [19,39]. In opposition to the SP, the VWBR possesses a vertically rotating wheel that occupies a large volume of the flask, providing a more gentle and homogeneous mixing with minimized shear stress [22]. Two cultures were performed and two secretomes were retrieved in both systems. Two secretomes were collected in the AlphaMEM culture medium (SP1 and VWBR1), and the other two secretomes were collected in the Neurobasal-A culture medium (SP2 and VWBR2).

Cells were successfully expanded in both systems and despite the attained differences in maximum cell numbers, cell metabolism was in accordance with what has been reported [31]. Moreover, cells retrieved from MCs at the end of culture in both systems retained the capacity for multilineage differentiation and the characteristic immunophenotype, except for cells from the first culture in SP (SP1). In fact, these cells showed the expression of CD80, which is a characteristic marker of activated B-cells, macrophages, and dendritic cells [40]. We can exclude the hypothesis that cells already expressed this costimulatory molecule before culturing because cells cultured in static conditions using the same culture conditions and the same cell donor did not display this marker [23,31,32]. Therefore, it should be the result of experimental error and must be confirmed in future studies. We observed that there were secretome profile variations between the secretomes produced in both systems. Consequently, in the conditions of our study, secretomes did not show the same capacity towards the induction of neurodifferentiation of hNPCs and neuroprotection in a *C. elegans* PD model based on the overexpression of α-synuclein.

The in vitro application of SP2 and VWBR2 secretomes led to the differentiation of human CNS-derived cells, which was shown by the expression of markers for both immature (DCX-positive cells) and mature (MAP-2-positive cells) neurons. The induction of neurodifferentiation can be supported by the identification in all secretomes of trophic factors and other molecules involved in neurogenesis, neuronal survival, and maintenance, such as stromal cell-derived factor-1 (SDF-1), BDNF, vascular endothelial growth factor (VEGF), platelet-derived growth factor (PDGF)-BB, glial cell line-derived neurotrophic factor (GDNF), and hepatocyte growth factor (HGF), among others [41,42,43,44]. Nonetheless, in the conditions of our study, SP2 secretome had a superior performance as it was able to increase the survival and differentiation of hNPCs into immature neurons, compared with VWBR2 secretome and control medium (Neurobasal-A medium). According to our results, the relative intensity of interleukin-4 (IL-4) cytokine was greater in the SP2 secretome compared to the VWBR2 secretome. This is particularly interesting because IL-4 was shown to induce neural stem/progenitor cell proliferation and neurogenesis when injected in healthy zebrafish [45].

The effects of the prolonged exposure to SP1 and VWBR1 secretomes in the survival of dopaminergic neurons overexpressing α-synuclein in a *C. elegans* model revealed that the secretome collected in SP had a superior neuroprotective potential. We have previously shown an apparently superior effect of the secretome from static conditions in the same model [30]. However, throughout the present work, we observed that control animals presented reduced neuronal loss. This was the reason for increasing the period for incubation with secretome from 7 to 10 days herein. We hypothesized that, under these conditions, characterized by an increased DA neuron death, the secretome might induce a greater neuroprotective effect. Based on our array analysis, MMP2 was not detected in VWBR1 and seems to be more prevalent in VWBR2 secretome compared with SP1 and SP2. As already mentioned, MMP2 was identified in the secretomes and considered a molecule involved in the cleavage of preformed fibrils of α-synuclein [46]. So, we hypothesize that the reduced expression of MMP2 in SP1 and absence in VWBR1 might be possible contributors to our results.

Although outside the scope of this work, the exploration of other biomolecules within the secretome, such as lipids and metabolites, that could have played a role in the effects herein witnessed is warranted [47].

The MSCs used in this work were isolated from the same tissue and the same donor and cultured in different dynamic systems. For each culture system, we employed previously established and optimized culture conditions aimed at optimizing cell expansion [22,23,31,32,33]. Therefore, the difference in culture conditions in both systems is significant, namely, the culture medium used in the cell expansion stage. Still, the culture medium used for conditioning and secretome collection was the same. Consequently, in this study, different elements should be considered as possible contributors to the different properties of the secretomes. It is noteworthy that the trophic factors identified here may be either present in the soluble fraction of the secretome or may be shuttled inside extracellular vesicles secreted by MSC. Further work may be developed within this scope to identify how MSC secrete these trophic factors and which secretome fraction is mainly responsible for the functional activities identified in the present study. Cells cultured in a mechanically agitated environment are subjected to hydrodynamic shear stress and the SP is reported to induce higher levels of shear stress, whereas the design of VWBR enables the reduction in agitation needed to maintain the homogenization of the system, contributing to lower shear stress [48]. Even though shear stress is known to impact MSC growth, it is still poorly known how the hydrodynamic shear modulates the secretory patterns of MSC [49]. In line with this, Diaz and co-workers showed that when exposed to fluid shear stress caused by vascular flow, BMSC immunomodulatory function is activated, suggesting that mechanical preconditioning of MSC could be an effective strategy to modulate the secretome [50,51]. Curiously, our analysis showed that IL-6, oncostatin M, transforming growth factor (TGF)-β1, and IL-4 (cytokines involved in the regulation of anti-inflammatory responses [52,53]) presented higher relative intensities in both secretomes produced in SP or at least in one of them.

The use of culture medium and other solutions containing animal-derived products, such as fetal bovine serum (FBS), for cell expansion purposes involves risks when the cells or their products are meant to be applied in the clinical setting. Indeed, these products do not have a defined number of components and present the risk of transmission of unknown infectious agents. Thus, large-scale expansion of MSC using defined culture media that is FBS-free is critical for translational research [54]. In this work, the culture medium used in VWBR cultures was a xeno-free medium supplemented with human platelet lysate [22] and the medium used in SP cultures was a commercially available xeno- and serum-free formulation [23]. A study that compared MSC grown in xeno- and serum-free conditions with an FBS-containing medium concluded that MSC grown in xeno- and serum-free conditions had a higher immunosuppression activity [54]. More recently, Yoshida and colleagues showed that serum-free culture conditions can improve the immunosuppressive capacity of MSC, which is aligned with our results [55]. However, this is a debatable subject because other studies suggested the opposite [56,57].

Another contrast between cultures in SP and VWBR was the concentration of glucose throughout the expansion period. The average concentration of glucose for SP cultures before conditioning was 3.4 ± 1.1 mM whereas for VWBR cultures was almost three times higher (9.5 ± 3.4 mM). There is evidence showing that the expansion of MSC in low (5.5 mM) and high (20 mM or 30 mM) glucose concentrations does not alter the secretion of VEGF, HGF, and basic fibroblast growth factor (bFGF) [58]. However, cells were subjected to those glucose concentrations for only 24 h and 48 h. So, the hypothesis that longer exposure to different concentrations of glucose might impact the secretion of specific factors cannot be ignored.

The results from the present work suggest that the culture conditions might have contributed to the distinct secretory profiles. Thus, more studies should focus on the effects of each dynamic system on the secretome profile using uniform protocols.

## 5. Conclusions

Overall, this study shows that the expansion of MSC in scalable dynamic culture can be employed to manufacture a cell-free product that can be used for repair strategies within PD models. Moreover, according to our results, the protocol/system can influence the MSC secretory profile and, consequently, the magnitude of its effect. This is particularly relevant given the important role of these types of systems in producing translatable products for clinical application.

## Figures and Tables

**Table 1:** Characteristics of BMSC expansion in both dynamic systems.

Dynamic System	Culture Medium Used for Cell Expansion	Culture Medium Used for Conditioning	Agitation Rate (rpm)	Cell Adhesion Efficiency (%)	Specific Growth Rate (Day^−1^)	Fold Expansion	Doubling Time (Day)
VWBR1	DMEM 5% UltraGRO™-PURE	AlphaMEM	30	76	0.31	4.19	2.24
VWBR2	DMEM 5% UltraGRO™-PURE	Neurobasal-A	25	31	0.24	2.58	2.90
SP1	StemPro MSC SFM	AlphaMEM	40	48	0.36	2.93	1.92
SP2	StemPro MSC SFM	Neurobasal-A	40	55	0.31	2.45	2.23

## Data Availability

The data presented in this study are available on request from the corresponding author.

## Supplementary Materials

The following supporting information can be downloaded at https://www.mdpi.com/article/10.3390/biomedicines11051240/s1, Supplementary Table S1: Statistical report. Supplementary Figure S1: Average yield of lactate from glucose (Y’Lac/Glc). The Y’Lac/Glc was determined throughout time for cultures in the (A) SP and in the (B) VWBR. Supplementary Figure S2: Gating strategy for the MSC markers.
