# Supplementary material for: Neurodifferentiation and Neuroprotection Potential of Mesenchymal Stromal Cell-Derived Secretome Produced in Different Dynamic Systems
Source: Biomedicines. 2023 Apr 22;11(5):1240. doi: 10.3390/biomedicines11051240 (PMC10215864; doi:10.3390/biomedicines11051240)

Supplementary Material for Biomedicines id:2223140

Article: Neurodifferentiation and neuroprotection potential of mesenchymal stromal cell-derived secretome produced in different dynamic systems

Cláudia Raquel Marques[1,2], Miguel de Almeida Fuzeta[3,4], Raquel Medina dos Santos Cunha[3,4], Joana Pereira de Sousa[1,2], Deolinda Silva[1,2], Jonas Campos[1,2], Andreia Teixeira-Castro[1,2], Rui Amandi Sousa[5], Ana Fernandes-Platzgummer[3,4], Cláudia Lobato da Silva[3,4†], António José Salgado[1,2†*]

1 Supplementary Table

Supplementary Table S1. Statistical report. Effect sizes calculated using Lenhard, W. (2016), Uanhoro, J. O. (2017), and https://www.psychometrica.de/effect_size.html. Effect size calculator available online at https://effect-size-calculator.herokuapp.com/.

Figure	Statistical report	Sample size
**4 B**	$F(2,155.566) = 29.911, p < 0.001, \omega^2 p = 0.267$	160 (per group)
**4 C**	$F(2,183.822) = 105.599, p < 0.001, \omega^2 p = 0.528$	160 (per group)
**5 A**	$F(3,73.496) = 24.690, p < 0.001, \omega^2 p = 0.478$	36 (per group)
**5 B**	$\chi^2\ (3) = 38.223, p < 0.001, V = 0.297$	36 (per group)
**5 C**	CEP $\chi^2\ (3) = 43.923, p < 0.001, V = 0.319$<br>ADE $\chi^2\ (3) = 17.542, p < 0.001, V = 0.201$	36 (per group)

2 Supplementary Figures

Supplementary Figure S1. Average yield of lactate from glucose ($Y'_{Lac/Glc}$). The $Y'_{Lac/Glc}$ was determined throughout time for cultures in the (A) SP and in the (B) VWBR. Results are presented as mean ± SD. SP, Spinner flask system; VWBR, Vertical-Wheel™ bioreactor; SD, standard deviation.

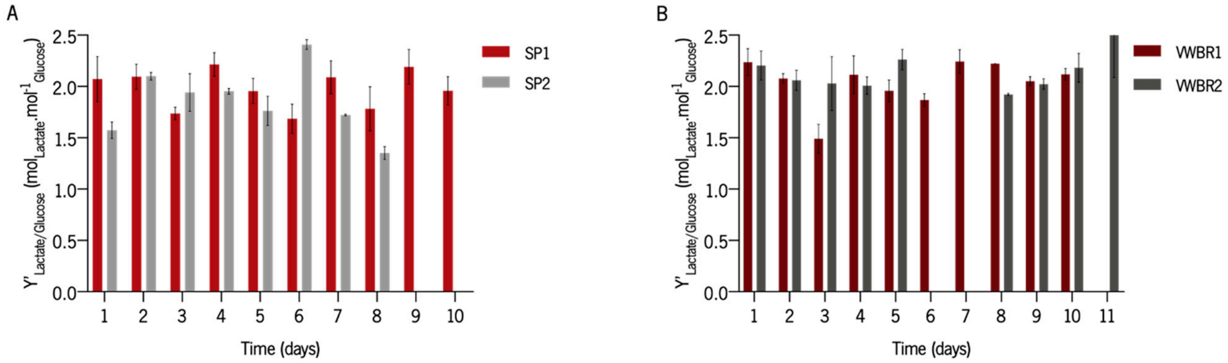

Supplementary Figure S2. Gating strategy for the MSC markers according to Dominici et al. 2006 [1]. Dot plots are representative from the VWBR cells. The same strategy was employed in all expansions systems. .

Marker	Positive %
CD14	9.28
CD19	4.63
CD34	3.54
CD45	4.27
CD73	99.3
CD80	5.67
CD90	97.3
CD105	97.4
HLA-DR	12.0

MSC negative markers
MSC positive markers

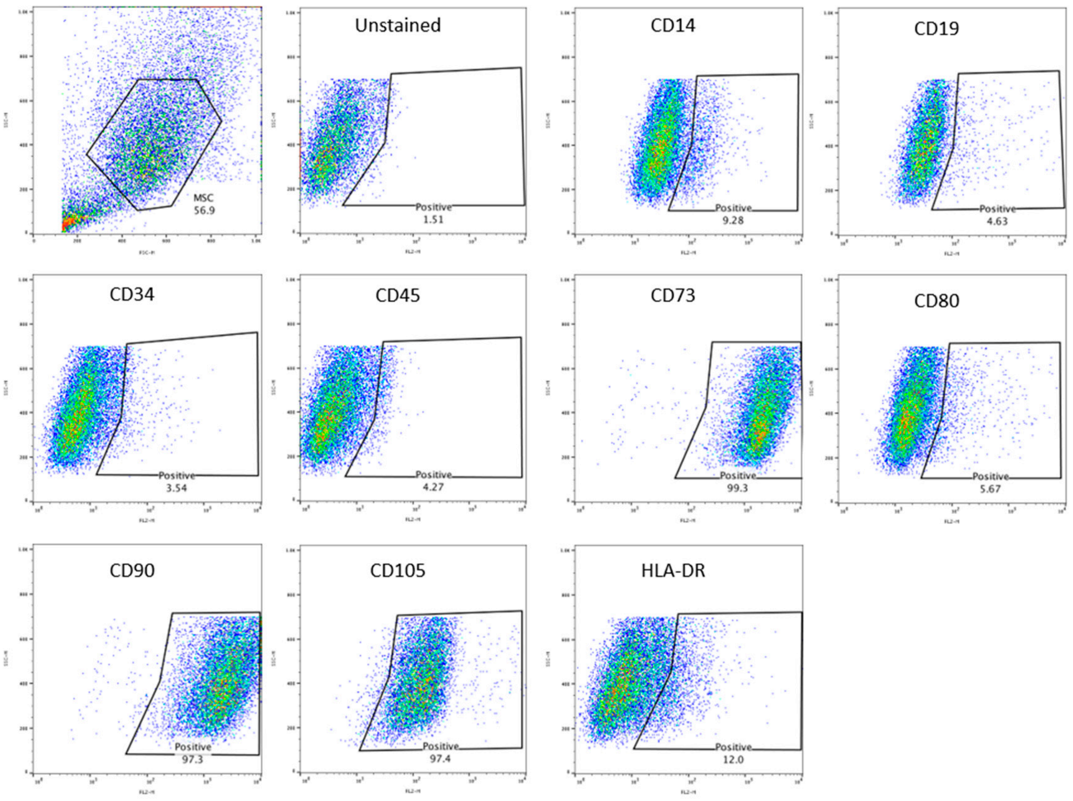

Supplement: Supplementary file 1 [file biomedicines-11-01240-s001.zip › biomedicines-2223140-supplementary.pdf]
